# Optimization of the Effect of Pineapple By-Products Enhanced in Bromelain by Hydrostatic Pressure on the Texture and Overall Quality of Silverside Beef Cut

**DOI:** 10.3390/foods9121752

**Published:** 2020-11-26

**Authors:** Diana I. Santos, Maria João Fraqueza, Hugo Pissarra, Jorge A. Saraiva, António A. Vicente, Margarida Moldão-Martins

**Affiliations:** 1 LEAF, Linking Landscape, Environment, Agriculture and Food, School of Agriculture, University of Lisbon, Tapada da Ajuda, 1349-017 Lisbon, Portugal; dianaisasantos@isa.ulisboa.pt (D.I.S.); mmoldao@isa.ulisboa.pt (M.M.-M.); 2 CIISA, Centre for Interdisciplinary Research in Animal Health, Faculty of Veterinary Medicine, University of Lisbon, Av. da Universidade Técnica, Pólo Universitário, Alto da Ajuda, 1300-477 Lisbon, Portugal; hpissarra@fmv.ulisboa.pt; 3 LAQV-REQUIMTE, Department of Chemistry, University of Aveiro, 3810-193 Aveiro, Portugal; jorgesaraiva@ua.pt; 4 CEB, Centro de Engenharia Biológica, Departamento de Engenharia Biológica, Universidade de Minho, Campus de Gualtar, 4710–057 Braga, Portugal; avicente@deb.uminho.pt

**Keywords:** pineapple by-products, hydrostatic pressure, bromelain, enzyme activity, marinade, meat, texture

## Abstract

Dehydrated pineapple by-products enriched in bromelain using a hydrostatic pressure treatment (225 MPa, 8.5 min) were added in marinades to improve beef properties. The steaks from the silverside cut (2 ± 0.5 cm thickness and weight 270 ± 50 g), characterized as harder and cheaper, were immersed in marinades that were added to dehydrated and pressurized pineapple by-products that corresponded to a bromelain concentration of 0–20 mg tyrosine, 100 g^−1^ meat, and 0–24 h time, according to the central composite factorial design matrix. Samples were characterized in terms of marination yield, pH, color, and histology. Subsequently, samples were cooked in a water-bath (80 °C, 15 min), stabilized (4 °C, 24 h), and measured for cooking loss, pH, color, hardness, and histology. Marinades (12–24 h) and bromelain concentration (10–20 mg tyrosine.100 g^−1^ meat) reduced pH and hardness, increased marination yield, and resulted in a lighter color. Although refrigeration was not an optimal temperature for bromelain activity, meat hardness decreased (41%). Thus, the use of pineapple by-products in brine allowed for the valorization of lower commercial value steak cuts.

## 1. Introduction

Bromelain is a proteolytic enzyme present in the *Bromeliaceae* family to which pineapple (*Ananas comosus* L.) belongs. Bromelain is also found in pineapple by-products (cores, peels, and leaves), though in lower amounts than in stems and fruits [1,2]. The minimally processed industry produces a large amount ~50% (*w*/*w*) of pineapple by-products [3]. These by-products remain metabolically active and respond to postharvest abiotic stresses that induce biosynthesis and accumulation on secondary metabolites. Hydrostatic pressure treatments (50–250 MPa), as abiotic stress, activate cellular processes [4]. Pineapple by-products are rich in compounds of interest (i.e., bromelain) that can be enhanced by the application of abiotic stresses via hydrostatic pressure, avoiding extraction technologies. Extraction usually involves large quantities of volatile and flammable organic solvents with negative impacts on the environment and economy. The application of a green technology could value by-products with potential application in the food industry. On the other hand, consumers demand processes without chemicals. Therefore, the food industry’s potential valorization of the pineapple by-products enriched in bromelain as a novel food ingredient allows for the possibility of consuming compounds that offer health benefits and reduce environmental impact [5].

The marinating process is a method that improves the sensory properties of meat. Marinating consists of immersing or injecting meat with a solution that may contain several ingredients, i.e., water, salts, vinegar, lemon juice, wine, soy sauce, essential oils, tenderizers, herbs, spices, and organic acids with the aim of flavoring and tenderizing meat or meat products [6]. Marinades are important in muscles rich in connective tissue and tenderizing properties offer means of commercial improvement [7,8]. Marinades develop an extra succulent texture on the surface of meat and reduce water loss during cooking. The soaking in marinades increases the tenderness of meat due to the increase in volume induced by the pH of the muscle fibers and/or connective tissue, faster proteolytic weakening of the muscular structure, and greater collagen solubilization after cooking [9].

The proteolytic enzymes present in fruits can degrade myofibrillar proteins and collagen in meat and, consequently, have beneficial effects on tenderness. Meat tenderized with proteolytic enzymes improves consumer acceptability, although the improvement in tenderization can be related with a decrease in yield [10]. The positive effect of proteolytic enzymes obtained from fruits on meat tenderness has been reported by some authors [11,12]. Cysteine proteases such as bromelain have been studied in relation to meat tenderization [13]. The amino acid structure of meat is crucial for the softening effect by bromelain because fragmentation occurs in lysine, alanine, tyrosine, and glycine [14]. Bromelain affects the structure of the myosin and actin filaments in myofibrillar proteins. Proteolytic enzymes increase the rate of myofibril fragmentation in meat and interrupt the intramuscular connective tissue structure [15,16]. Bromelain is active against collagen proteins [12]. Bromelain has maximum enzymatic activity in the temperature range of 37–70 °C [17]. At refrigeration temperatures, the enzyme has low activity, although it is active at 0 °C [18].

The USDA Food Safety Inspection Service currently considered the enzymes bromelain, papain, ficin, *Bacillus* proteases, and aspartic proteases to be safe [12]. Proteolytic enzymes extracted from plants, namely papain, bromelain, actinidin, zingibain, and ficin have been widely used as meat tenderizers in several countries [13,19].

The present work aimed to optimize the effects of marinade with pineapple by-products (enriched in bromelain through treatment with hydrostatic pressure) at different concentrations and contact time on the sensory characteristics (particularly the hardness) of steaks from silverside cuts considered to have a lower commercial value due to its sensory characteristics.

## 2. Materials and Methods 

### 2.1. Meat Samples Collecting and Preparation

The beef cuts silverside (lot 332199042) were from animals that were born, raised, slaughtered, deboned, cut, and vacuum packaged in Poland according to EU standard commercial practices. Further, they were transported under refrigeration (0–4 °C) conditions to Portugal where they were again labelled (lot 194948). Two independent beef cuts (silverside cut constituted mainly by *M. gluteobiceps and M. semitendinosus*) from different bovines (Piece 1: 4715 g and Piece 2: 4225 g) were supplied by a central logistic of hypermarkets in Santarém, Portugal, and transported under refrigerated conditions (0–4 °C) to the Faculty of Veterinary Medicine, University of Lisbon. The assay was performed 10 days after the animals slaughtering. Each beef cut was sliced with a sharp knife into 16 steaks with 2 ± 0.5 cm thickness and a weight of 270 ± 50 g.

### 2.2. Production of Bromelain Powder from Pineapple By-Products

Pineapple (*Ananas comosus* L.) by-products were provided by the company Campotec S.A. located in Torres Vedras, west center of Portugal. The core pineapples (~104.5 × 30 mm) were stored under refrigeration (5 ± 1 °C) approximately 3 h prior to packaging in PA/PE-90 (Alempack - Embalagens Flexíveis, Elvas, Portugal) that were vacuum sealed (85% of vacuum).

An abiotic stress in packaged by-products (pineapple core) was applied using a pilot-scale high pressure hydrostatic equipment (Hiperbaric 55, Burgos, Spain) with a 55 L vessel, according to the conditions optimized in a previous work (225 MPa during 8.5 min) [20]. The core pineapple samples after pressurization were stored at 5 ± 1 °C for 24 h. After storage time all samples were frozen at −80 °C and lyophilized (Coolsafe Superior Touch, Scanvac, Denmark) during 7 days at −94 °C and 0.2 to 0.09 mbar. Further, 300 g samples were ground in a food processor (Bimby Thermomix TM31, Vorwerk Thermomix, Cloyes-sur-le-Loir, France) at a speed of 10,200 rpm for 25 s to obtain the powder sample. The enzymatic activity of bromelain was quantified in the pineapple powder core sample and showed 9.33 ± 0.29 mg tyrosine. g^−1^ dry matter according to Chakraborty (2014) with some modifications [21].

### 2.3. Marinating

#### 2.3.1. Optimization of Pineapple By-Products Enhanced in Bromelain Application on Marinated Steaks

Response surface methodology (RSM) constructed on a two-variable central composite rotatable design (CCRD) was used as a function of two factors [22]. The experimental data through a stepwise multiple regression analysis using StatisticaTM v.8 Software (StatSoft Inc., Tulsa, OK, USA, 2007) was fitted to a second-order polynomial equation to predict each dependent variable (*Y*). The three-dimensional response surface designs as a function of independent variables (bromelain concentration (*X*_1_; mg tyrosine.100 g^−1^ meat) and time (*X*_2_; min)). *b*_0_ is the interception and *b*_i_, *b*_j_, and *b*_ij_ (i,j = 1,2) are the linear, quadratic, and interaction coefficients, respectively, that are described by the second order polynomial models, using decoded variables, as follows Equation (1).

(1) $$Y_{i}=b_{0}+b_{1}X_{1}+b_{2}X_{2}+b_{11}X_{1}^{2}+b_{22}X_{2}^{2}+b_{12}X_{1}X_{2}$$

A brine was prepared by dissolving 1% (*w*/*w*) of coarse salt (purified sea salt for seasoning and cooking, Pingo Doce: Lot AL73527697) in water and stored at 4 ± 1 °C for 3 h to stabilize. Each steak was placed in a container (23 × 16 × 5.5 cm) and the brine was added.

The pineapple powder core (0 to 20 mg tyrosine.100 g^−1^ meat) was suspended in 400 mL of brine according to the experimental design matrix in Table 1. The brine mixture with pineapple powder core was then added to the container with the meat, and the meat remained immersed at 4 ± 1 °C for the time (0 to 24 h) defined by the experimental design for each sample.

The samples were weighed, the color and pH of the steaks was determined, and the pH of the brine were evaluated before marinating. After the marinade, the samples were weighed again, the color and pH of the steaks was determined, and the pH of the brine were evaluated, and the sample was collected for the histological analysis. Subsequently, the steaks were packaged in PA/PE 90 µm (polyamide (PA) 20 µm, polyethylene (PE) 70 µm provided by Luis Sanchez and Filhos Lda., Sintra, Portugal) that were vacuum sealed (85% vacuum - Henkovac, quality vacuum systems, De Brand, Netherlands). The samples were prepared in duplicate for each treatment of the experimental design.

#### 2.3.2. Brine Effect on Marinated Steaks

In order to study the effect of adding brine, marinades were performed without adding pineapple powder over time (0–24 h). In the control sample, the steak was placed in the container and the brine 1% (*w*/*w*) was not added (0 h). Brines 1% (*w*/*w*) without the addition of pineapple powder core were added to the steak to assess the effect of the brine on steak over time (3.5 h, 12 h, 20.5 h, and 24 h).

### 2.4. Cooking

The cooking process was carried out in a water bath set at 80 ± 1 °C, in which the vacuum-packed meat (4 ± 1 °C) was added. When the temperature of the water bath achieved 80 ± 1 °C again, the cooking process started and lasted for 15 min in order to reach 75 ± 1 °C at the coldest point (center of the steaks). Subsequently, the steaks were placed in a water and ice bath for 30 to 40 min until reaching 4 ± 1 °C in the slowest cooling point (center of the steak) [23]. The cooking temperature and the cooling of the steaks were monitored by thermocouple (*Ellab* a-s model CTF 9008, Copenhagen, Denmark). After cooking and cooling, the samples were stored for about 24 h at 4 °C to stabilize the characteristics of the steaks. Subsequently, the samples were weighed again, the color and pH of the steaks were evaluated, the texture was determined, and samples were collected for histology.

### 2.5. Analytical Methods

Each steak sample subjected to each treatment was used to analyze the margination yield, cooking yield, pH, color, texture, and histology. The standard deviation in the results indicates the variation between the two pieces of meat and the repetitions of the analytical method.

#### 2.5.1. pH Measurement

The pH values were measured with a digital pH meter (Hanna Instruments, Woonsocket, RI, USA). The pH meter was calibrated with pH 7 and 4 buffers before pH determination. The stainless-steel blade was fitted to the solid sample probe to facilitate penetration into the meat. Cutting the meat allowed direct contact between the probe and the sample, so that the pH was measured directly without further sample preparation. The initial pH of the steaks was measured 2 h after opening the vacuum bag and the initial pH of the brine was measured 3 h after preparing the solution. The pH of the steaks and brine was measured again after the marinating time of each sample. After cooking (24 h), the pH of the steaks was determined three times on each sample [24].

#### 2.5.2. Measurement of Marination Yield

The initial weight of the steaks was determined immediately after cutting using an analytical balance (Sartorius A200S, Göttingen, Germany). After the marinade time, the steaks were removed from the brine, dried with paper towels to remove superficial water (blotted drying), and weighed again. The marination yield (MY) was calculated by the difference between raw and marinated weights as following: marination yield (%) = ((weight of marinated steak − weight of raw steak)/weight of raw steak) × 100.

#### 2.5.3. Measurement of Cooking Yield

The meat samples were weighed just before cooking. The steaks after 24 h of stabilization at 4 °C after cooking were removed from the bag, dried with paper towels to remove excess surface moisture, and reweighed using an analytical balance. The cooking yield was calculated by the difference between raw and cooked weights as following: Cooking yield (%) = (weight of cooked steak/weight of raw marinated steak) × 100 [23].

#### 2.5.4. Color Measurement

The color was measured on the surface of steaks with a Minolta CR 300 (Konica Minolta, Osaka, Japan) using the L*, a*, b* coordinates (CIELab color system). The measurement Minolta CR 300 uses diffuse illumination/0° viewing angle and aperture sizes of 8 mm. The calibration was performed with a white ceramic reference (standard illuminant D65). The initial color of the steaks was measured 2 h after opening the vacuum bag of the piece of meat, after the marinating time associated with each sample and 24 h after cooking the steaks. The color was measured 3 times on each sample and each value resulted from the arithmetic mean of three measurements. The results are presented in terms of variation (Δ) in relation to the initial color for marinated samples. In the case of cooked samples, the color variation (Δ) was calculated in relation to the unmarinated cooked sample.

#### 2.5.5. Mechanical Texture Measurement

The texture properties of the steaks were analyzed with a texture analyzer TA. XT plus (Stable Micro Systems, Surrey, UK) equipped with a Warner–Bratzler V-shaped blade (0.9 mm thickness and a triangular aperture of 60°). The sample was cut into small pieces of known size with parallelepipedal form (2 × 1 × 1 cm). The texture analyzer was calibrated with a 5 kg load cell. The steaks were cut perpendicular to the longitudinal muscle fibers. The Warner–Bratzler blade was pressed down (compression test mode) at a constant pre-test and test speed of 5 mm.s^−1^, and post-test speed of 10 mm.s^−1^ through the sample and the maximum shear force (Newton) were recorded. The texture of the steaks was determined twenty times on each sample [23,25].

#### 2.5.6. Histological Technique

For histological analysis, a small portion of a steak (about 2 × 2 × 1 cm) was collected from each treatment’s sample (marinade and cooking) and control (raw steak). These samples were placed individually in plastic cups with 10% neutral formalin until the histological processing took place. The sample cuts must be fine to facilitate the fixing of the formol (thickness not exceeding 1 cm). These samples were fixed in 10% neutral buffered formalin and submitted for histological routine processing technique (fixation in formalin, embedding in paraffin, and staining with hematoxylin and eosin). The slides were observed under an optical microscope, and photographs of the histological sections were made with an Olympus DP21 digital camera [26].

#### 2.5.7. Statistical Analysis and Model Fitting

The experimental data was statistically evaluated through the StatisticaTM v.8 Software (StatSoft Inc., 2007, Tulsa, OK, USA) from Statsoft (StatSoft Inc., Tulsa, OK, USA) [27]. The multiple regression analysis was fitted to a second-order polynomial equation to predict each dependent variable (pH, marination yield, cooking yield, color, and hardness of steaks). The three-dimensional response surface designs are described by the second order polynomial models, using decoded variables, as a function of independent variables bromelain concentration on the brine (mg tyrosine.100 g^−1^ meat) and contact time (h). The adequacy of the model to fit the experimental data was verified by the analysis of variance (ANOVA) and coefficient of determination (R^2^) and adjusted R^2^ (Adj-R^2^) [22].

For the tested parameters, obtained results were compared to those of the same contact times of the experimental design. Tukey’s honest significant difference (HSD) test was used to determine the significant differences among means for different treatments. The accepted level of significant differences was *p* < 0.05.

## 3. Results

### 3.1. Optimization of Pineapple By-Products Enhanced in Bromelain Application on Marinated Steaks

#### 3.1.1. pH Value

**Figure 1:**
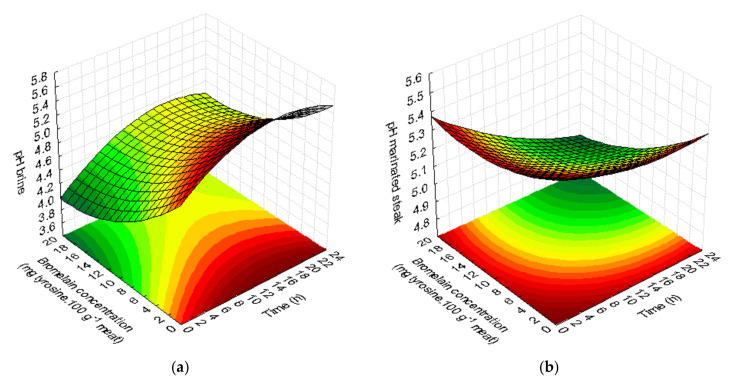
Effect of marinades on pH as a function of bromelain concentration (mg tyrosine.100 g^−1^ meat) and time (h). Response surfaces fitted to pH of brine (**a**) and pH of marinated steak (**b**).

The addition of greater amounts of bromelain to the brine promoted a decrease in pH, making the brine more acidic (the initial pH of steak was 5.44), as can be seen in Figure 1a. The surface response methodology described very well the pH change that occurs over time at the different concentrations of bromelain introduced in the marinade. The model presented a very good fit, with R^2^ = 0.97 and Adj-R^2^ = 0.94 and the adequacy of the second-order polynomial model (Table 2) as confirmed by the not significant lack of fit (*p* > 0.05). According to the model, the variables time and concentration of bromelain significantly influence the marinade pH values. Marinades with higher amounts of enzyme showed lower pH values (pH = 4.31–4.69). The lowest enzyme concentrations showed higher pH values (pH = 5.49–5.67) regardless of the marinating time (Figure 1a). Marinades with bromelain concentrations greater than 10 mg tyrosine.100 g^−1^ meat had pH values below 5.

The quadratic model described the behavior of pH in steaks after marinade (Table 2) with good fit (R^2^ = 0.95 and Adj-R^2^ = 0.92). Both bromelain concentration and immersion time had a linear negative significant effect. When the bromelain quantities were smaller, but the immersion time is longer (greater than or equal to 12 h), the steak pH decreased (Figure 1b). Samples with no added enzyme (0 mg tyrosine.100 g^−1^ meat) with small amounts (3 mg tyrosine.100 g^−1^ meat) for a short time (3.5 h) or 10 mg tyrosine.100 g^−1^ meat for a very short time (0 h), showed significantly higher pH values (*p* < 0.05) than steak samples marinated with higher concentrations of bromelain enzyme (17–20 mg tyrosine.100 g^−1^ meat). The pH values of treated steaks range from 4.99 to 5.44, with the highest value corresponding to the initial pH of the steak (without marinade). The lowest pH values were obtained for marinated steaks with 17 mg tyrosine.100 g^−1^ meat for 3.5 h (pH = 4.31) and 20.5 h (pH = 4.60) (*p* < 0.05).

The quadratic model did not adjust to the pH values of the steaks after cooking. The pH values fluctuated between 5.65 and 5.99.

#### 3.1.2. Marination Yield

**Figure 2:**
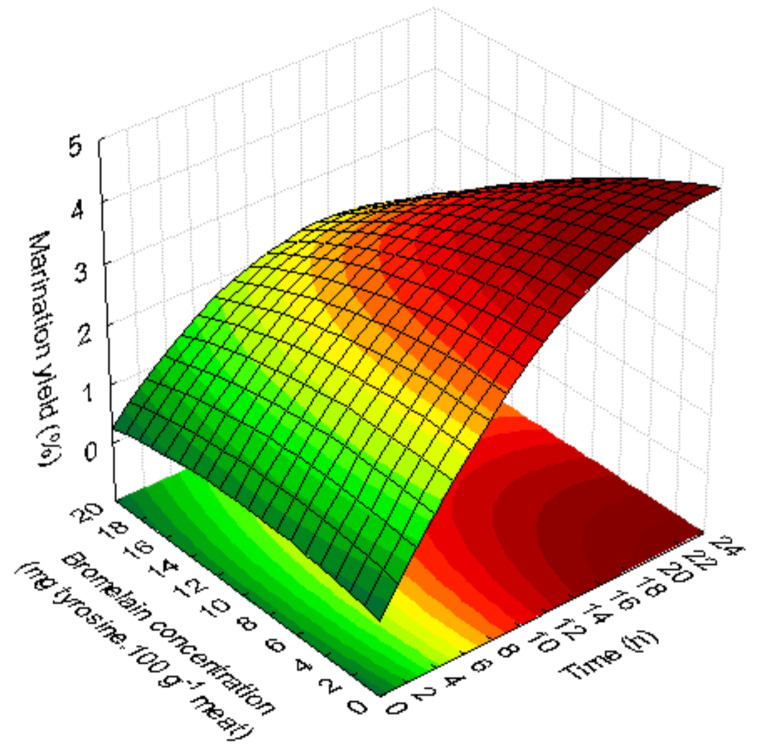
Effect of marinades on marination yield of the steaks as a function of marinades: bromelain concentration (mg tyrosine.100 g^−1^ meat) and time (h).

The quadratic model generated for the marination yield (MY) was significant in fitting the experimental data to a 95% confidence level. The R^2^ and Adj-R^2^ values were high (Table 2) and indicated a good fit to the data. In addition, the suitability of second-order polynomial models was validated by the lack of fit non-significant (*p* > 0.05). Steak samples had a MY of 0.07% to 4.19%. Only the linear positive significant effect of immerging time was observed. Figure 2 shows that longer times (12–24 h) increase the MY of the steak twice. Longer times (20.5–24 h) and low or intermediate bromelain concentrations (0–10 mg tyrosine.100 g^−1^ meat) increased steak MY, while shorter times (0–3.5 h) and intermediate or high concentrations (10–17 mg tyrosine.100 g^−1^ meat) of enzymes decreased MY.

#### 3.1.3. Cooking Yield

The generated model did not have a good fit and the study variables (bromelain concentration and time) did not have a significant influence on cooking performance (Table 2).

#### 3.1.4. Color

The obtained models for the color parameters show that the color is significantly influenced by the immersion time in brine and that the concentration of bromelain does not have any significant effect on this parameter. The effect of the time during which meat was in the brine on ΔL* was more relevant than the effect of bromelain concentration (Table 2).

The values presented in response surface methodology are related to the difference between the marinated samples and initial values of color. The quadratic models generated for the values of L for both marinated and cooked steaks were significant in fitting the experimental data to a 95% confidence level, and the R^2^ and Adj-R^2^ values were high (Table 2). The suitability of second-order polynomial models was validated by the lack of fit non-significant (*p* > 0.05). With respect to the L* parameter of the marinated steaks and cooked steaks, linear positive and quadratic negative significant effects (*p* < 0.05) of the marinating time were observed.

**Figure 3:**
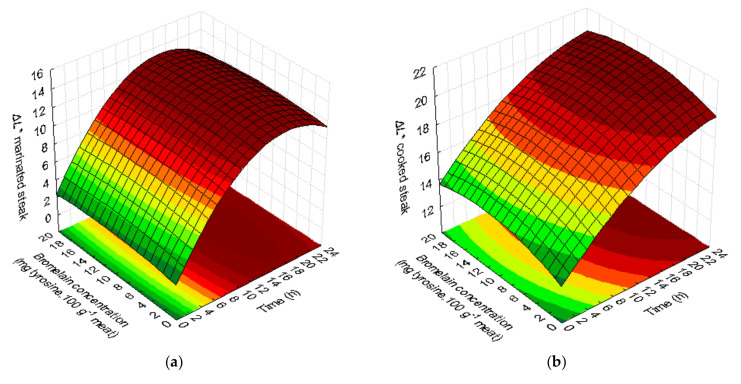

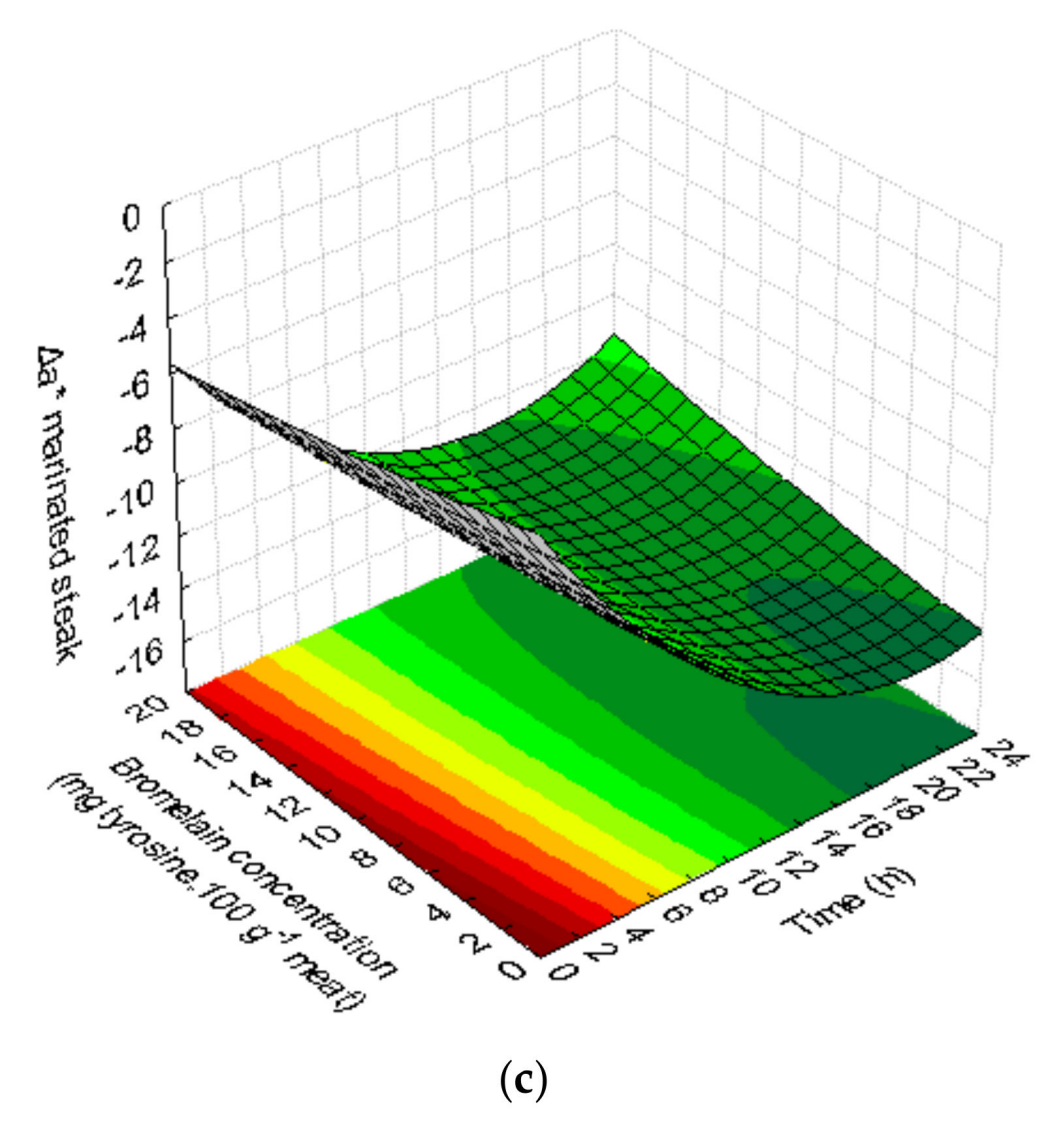
Response surfaces fitted for color parameters in marinated steaks: parameter ΔL* (**a**), parameter Δa* (**c**), and parameter ΔL*in cooked steaks (**b**).

The second-order polynomial model generated for ΔL* shows that higher ΔL* values for marinade steaks were observed between 12–22 h, regardless of the bromelain concentration (Figure 3a).

Figure 3b shows that an increase in the value of parameter ΔL* of steaks after cooking was observed after 15 h of marinade, regardless of the concentration of bromelain. Time is the significant variable in the model, while the bromelain concentration has no significance in the model. It was observed that longer times (>12 h), with or without the addition of the enzyme, made the steaks lighter/paler after cooking (*p* < 0.05).

The color parameter a* (Table 1) showed significantly higher values (21.21) for bromelain concentrations of 10 mg tyrosine.100 g^−1^ meat, with shorter marinade times (0 h). The longest marinades (24 h) with the addition of bromelain enzyme (10 mg tyrosine.100 g^−1^ meat) had the lowest value of parameter a* (11.47). After cooking, steaks without marinade had a* value of 10.16 and the longest marinade (24 h) with addition of enzyme (10 mg tyrosine.100 g^−1^ meat) induced the lowest a* value on steak (6.42).

The model obtained for parameter Δa* of the marinated steak presents a good fit to the data (R^2^ = 0.86 and Adj-R^2^ = 0.74) and a not significant lack of fit (Table 2). Figure 3c shows that the highest values of Δa* were obtained for shorter times (significant variable), regardless of the concentration of bromelain in the marinade. Parameter Δa* emphasizes the loss of meat pigments, becoming less red due to the loss of myoglobin.

The quadratic models obtained for color parameters Δa* and Δb* after steak cooking have insufficient adjustment to the data (R^2^ < 0.75) and therefore are not shown. Cooked samples with shorter marinating time showed lower Δa* values and, as for ΔL* values, the variable that influenced the results was time. 

The quadratic model generated for the color parameter Δb* of the marinated steak did not fit and did not present a well-defined tendency (R^2^ < 0.75). The color values obtained for the marinated steaks (3.26–6.32) did not show significant differences (*p* > 0.05) in color parameter b*. After cooking, the color values for parameter b* (8.75–11.99) also showed non-significant differences (Table 1). The steak sample with shortest time of contact with the marinade presented the highest a* and the lowest L*, in other words, they presented a color similar to the non-marinated steak (raw).

#### 3.1.5. Texture (Hardness)

**Figure 4:**
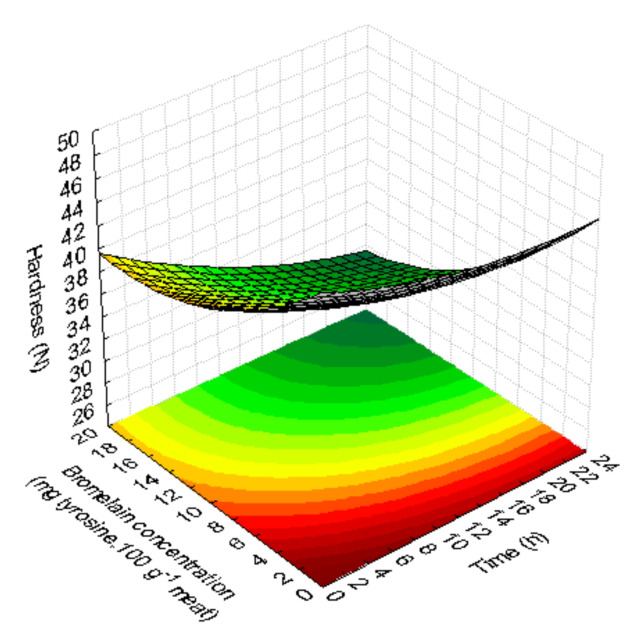
Response surface fitted for hardness as a function of marinades (bromelain concentration (mg tyrosine.100 g^−1^ meat) and time (h)).

The quadratic model obtained for hardness after cooking have adjustment of the data range (R^2^ > 0.75). The model showed a tendency of hardness to decrease for higher marinating times and higher concentrations of bromelain (Figure 4). Steaks that were not marinated had a significantly higher hardness (*p* < 0.05) than marinated steaks. Samples with a higher concentration of bromelain (17 mg and 20 mg tyrosine.100 g^−1^ meat) showed a tendency towards less hard steaks, although longer marinades (>12 h) with lower bromelain concentrations (3 and 10 mg tyrosine.100 g^−1^ meat) also reduced the hardness (Table 1).

#### 3.1.6. Evaluation of Histological Technique

The images obtained through the histological technique can be seen in the Table 3. Skeletal muscle fibers appear to be more affected by marinating time than by the concentration of bromelain. The skeletal muscle fibers suffered a slight degradation for the marinade with short times (3.5 h) and a moderate degradation for higher marinade times (12 h), although the concentration of bromelain was lower (10 mg tyrosine. 100 g^−1^ meat) in the marinade lasting 12 h than in the 3.5 h (17 mg tyrosine. 100 g^−1^ meat) marinade.

The cooked sample showed severe disorganization in the skeletal muscle fibers. These structural changes in skeletal muscle fibers occurred due to heat treatment.

The intermuscular collagen fibers presented a moderate degree of degradation, regardless of the marinade time and the concentration of the bromelain enzyme. The complete disorganization of the inter-muscular collagen fibers was observed for the cooked samples and not for the raw sample, with the same marinating conditions. It can be concluded that the disorganization of the inter-muscular collagen fibers is an effect of the heat treatment and not an effect of the conditions of the marinade.

Muscle fibers appeared swollen in the sample with the highest concentration of bromelain enzyme in the marinade, while for lower concentrations in the marinade the muscle fibers remain generally preserved.

### 3.2. Brine Effect on Marinated Steaks

**Figure 5:**
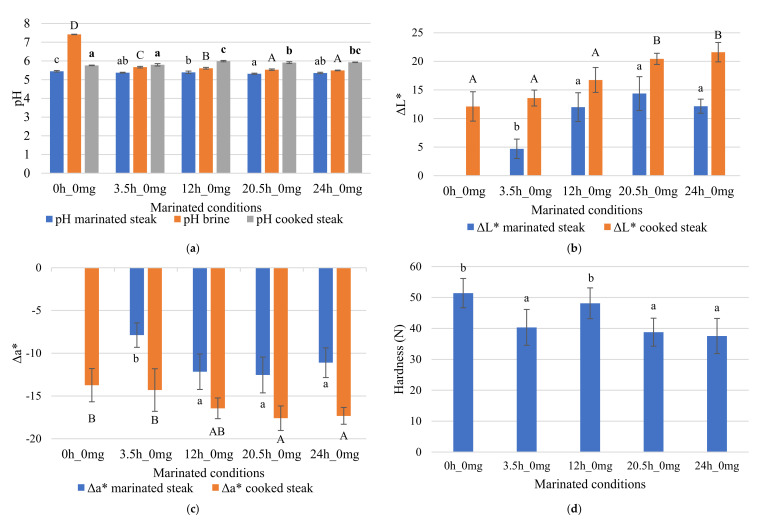
Study of the effect of adding brine on marinated steaks: (**a**) pH, (**b**) ΔL* color, (**c**) Δa* color, and (**d**) hardness. Error bars represent ± standard deviation (pH and color: *n* = 3 and hardness: *n* = 20). pH: Different letters express significant differences between pH marinated steak samples (lower case letters); between pH brine samples (upper case letters); and between pH cooked steak samples (bold lower-case letters). Color: Different letters express significant differences between color marinated steak samples (lower case letters) or between color cooked steak samples (upper case letters).

The initial brine (without addition of enzymes) showed a higher pH (7.42) and significantly different (*p* < 0.05) from the remaining brines (Figure 5a). The time (3.5 h, 12 h, 20.5 h, 24 h) showed a significant effect (*p* < 0.05) on lowering the pH of marinated steak and the pH of brine. We observed an increase of the cooked steak pH compared to the initial steak and brine pH (Figure 5a). 

A 24 h marinating time did not significantly influence MY (2.11–3.58%) when steaks were immersed just in brine.

The steak that was not marinated (control sample) showed a higher cooking yield (82.04%) than the marinated samples. The contacting time between steak and brine without bromelain enzyme imparted a reduction in cooking yield of 8.53% to 12.40% (69.64–73.51%).

The steaks without marinade showed the lowest value of L* (38.14), while the highest value of L* (52.45) was obtained for samples marinated for 20.5 h without adding enzymes, thus verifying the significant effect (*p* < 0.05) of the marinade time (Figure 5b). The L* parameter of the marinated steaks (Table 2) was significantly influenced by the marinating time (*p* < 0.05). After cooking, the untreated steak presented the lowest L* value (50.26) while samples marinated for 24 h showed the highest L* values (58.66) for a bromelain concentration of 0 mg tyrosine.100 g^−1^ meat. The color parameter ΔL* in marinated steak samples was significantly higher for samples with marinating time equal to or greater than 12 h. In cooked samples, the parameter ΔL* was significantly higher for marinades 20.5 h and 24 h, compared to shorter times (Figure 5b).

The marinated steak samples showed significantly lower Δa* values for marinades greater than 12 h and the cooked steak samples showed lower values for marinating times greater than 20.5 h (Figure 5c).

The immersion of steaks in brine seems to promote a reduction in hardness. Samples with marinade times 3.5 h, 20.5 h, and 24 h showed significantly lower values than samples without marinade (Figure 5d). The brine was advantageous in reducing the firmness of the steaks.

## 4. Discussion

The pineapple by-product is acidic and lowers the pH of the meat, which consequently increases the marination yield and lowers cooking yield. The pH of the brine significantly reduced to pH values below 5, with the increase in the concentration of bromelain, and for this reason the pH of meat was decreased with consequent denaturation of proteins, including possibly myoglobin. These changes on meat pH may have implications for its structure with influence on MY and cooking yield.

The enzymatic activity of bromelain in steak tenderization, besides being affected by immersion time and bromelain concentrations, is also affected by marinade pH and temperature [28,29]. The pH was reported to decrease in steak, chicken, and squid samples, after marinades with bromelain were at room temperature [30].

A slight change in pH can affect meat stability, and the pH promotes myoglobin oxidation. The pH value of meat greatly impacts MY, tenderness, and juiciness. The pH value of steak meat decreased after treatment with commercial pineapple stem bromelain and the greatest reduction was observed for steak meat treated with purified core bromelain [31].

Besides the pH of meat, the enzymatic action of bromelain on the protein structure can influence the marination yield. The fibers in the muscle are broken and lose their ability to retain water. Higher enzyme concentrations reduce the marination yield and, consequently, the meat loses water. MY is a crucial quality characteristic for sensory attributes, but also particularly important economically. The meat’s ability to retain its own water is an important feature of meat quality and contributes to its juiciness, flavor, and color [32]. The longer the steaks are in the brine, the greater the water absorption since the salt can promote protein solubilization and adsorb water. The addition of salt increased the MY of the meat, with a high MY due to a low interaction between actin and myosin [33]. The reduction in pH also influences the structure of myofibrillar proteins (principally myosin and actin) [34,35].

The salt (sodium chloride) changes the isoelectric point of the meat proteins to lower pH values and, consequently, changes the properties of the meat tissues, thus increasing water binding [36]. Salt increases the solubility of meat proteins and also increases ionic strength, which influences the increase of MY [37,38]. Sodium chloride (0 to 2%) applied to steak decreases the meat drip and increases the marination yield [38]. The marination yield is related to the myofibrillar protein, namely the proteins and structures that bind and entrap water. Myofibrillar proteins and myofibrils and muscle cells can entrap water, but this depends on the influence of pH, ionic strength, and oxidation [39]. MY is related to water structure and ion bonding. The binding of sodium and chloride ions in proteins and the salt-protein interactions in the water structure may explain the effect of salt on water retention through the selective binding of chloride ions in the hydrophobic regions of the myosin filaments [40]. The hydration force consists of binding the ion to the surface of the protein and forming the water around the ion creates a repulsive barrier [41].

Other authors have also referred the behavior of steak under bromelain marinade observed in this study. The reduction in MY depended on the influence of bromelain on the denaturation of myofibrillar proteins through the hydrolysis of tissue fibers [30,42].

The MY tendency can maintain, decrease, or increase. Some studies using enzyme treatments reported that the increases were not significant, while others described significant increases in MY in buffalo meat, steak, and turkey meat [43,44,45]. Other studies registered MY reductions in steak, chicken, and squid [32]. The reduction in the pH of meat from egg-laying chickens also influenced MY [46]. A study carried out in the post mortem of pork meat also observed the relationship between the decrease in pH, the mobility of myowater and related to the MY of the meat [35]. Another study demonstrated that the application of NaCl in steak increased the water retention capacity in the meat, which also reduced losses during cooking [47].

A higher enzyme concentration and a longer marinating time influenced the loss of cooking yield because the enzymatic action allowed for the alteration of cellular structure and reduced the capacity to retain water; on the other hand, the marinade with low salt concentrations allowed for MY to increase. The incorporation of NaCl (0.7% or 1.2% *w*/*w*) and cooking at temperatures (60–65 °C) increased the MY of muscles (*M. gluteobiceps*) with reduced weight loss during cooking. Meat treated with salt (NaCl) showed an increase in protein solubilization that may be related to the elimination of myofibrillar proteins and act as an impediment to the extraction of myosin. The softening of the protein structure and the conformational changes of myofibrillar proteins would allow water to be retained inside the tissue [48]. The elimination of water increased with an increase in cooking temperature and the contraction of myofibrillar proteins due to the increase in temperature for the period of warming, which influenced the results of cooking loss [49].

Meat quality and cooking yield are an industry concern, with repercussions also in meat attributes that affect consumer satisfaction and depend on the MY and meat pH. Cooking losses are reflected in meat weight loss. The results of this study were consistent with the results found by other authors. Weight loss due to cooking increased in samples marinated that contained pineapple juice over marinating time in egg-laying chicken samples [46]. The bromelain powder produced from pineapple increased the cooking loss of steak round cuts [15].

The cooking yield of steak, chicken, and squid samples treated with bromelain was lower than the control samples and it was also found that the cooking performance decreased with the increase of the bromelain extract [30]. In the present study, it was seen that the addition of bromelain promoted a decrease in the pH value of the meat, which caused less water retention and, consequently, lower cooking yields.

The analysis of the color values obtained in these assays indicates that the time the meat is in the brine is more relevant than the concentration of bromelain added to brine, in the color parameter ΔL. The steaks becoming paler can be explained by the solubilization of myoglobin in the brine during the marinade and, consequently, the concentration of myoglobin in steaks decreases. The changes observed in parameter Δa emphasize the loss of pigments in the color of the meat dipped in the brine.

Steak samples marinated with higher concentrations of bromelain have a lower pH and a lighter color with less red color. A study with bovine *Longissimus dorsi* muscles observed that the addition of citric acid in a brine at 2% NaCl (*w*/*v*) caused steaks to become lighter and less red in color than the brine at 2% NaCl (*w*/*v*). One of the reasons for this is that the lower pH in the citric acid solution influences the denaturation of muscle proteins and causes a greater reflection of light. The effects of marinades on luminosity and redness may result from effects on pH or ionic strength. The low pH also favors the oxidation of myoglobin [50].

A study with *semitendinosus* steak evaluated the purged liquid and found that the samples treated with enzyme injection (pancreatin) had a larger volume of liquid and had a darker red color in purged fluid. The author suggested that the weight lost in these samples signified water-soluble sarcoplasmic proteins that contained myoglobin. The steaks treated with enzymes showed a lighter color, probably due to loss of myoglobin [51].

Another study reported a decrease in luminosity (L* value) and an increase in redness (a* value) in the steak meat cut samples treated by the dipping method with purified bromelain reconstituted in distilled water compared to the control sample. These results were the opposite of the results obtained in this study, but the author mentions possible explanations for the observed color changes. Reducing the amount of metmyoglobin to myoglobin can improve color stability, since oxidation of myoglobin maintains the red color of meat. Lipid oxidation and pigment oxidation in steak are also associated with meat discoloration [31]. Bromelain being reconstituted only in distilled water and without adding sodium chloride may be an explanation for the differences observed. Moreover, the facts that the meat was put in contact with marinade for only 2 h at 25 °C with purified bromelain could explain why the meat was not paler. Furthermore, the initial pH meat value (6.1) was higher than the values reported in the present study (pH = 5.44) and, consequently, the behavior of the meat may not be the same.

The marinade with pineapple juice showed changes in color parameters in chicken meat after cooking [46]. Changes in the color of the meat can be caused by heat treatment [52]. The increase in temperature during cooking makes the meat paler (increases the L* value) and reduces the red color (decreases the a* value) [53].

The results obtained by other authors agreed with the results obtained in this study. The values of L * and b * increased for steak round cuts samples treated with bromelain powder [15].

Many studies of marinated meat concluded that marinades containing pineapple juice had the greatest impact on meat tenderness and caused the most intense structural and textural alterations, although the attractiveness of the meat worsened [8,9]. Bromelain has been extensively studied by several authors as meat tenderizer for various animals such as steak, mutton, chicken, and pork [30,54,55]. Pineapple peel bromelain was an efficient softener for the steak, chicken, and squid [30]. The samples of steak, chicken, and squid muscles treated with bromelain extract showed lower firmness (shear force values decreased) with an increase of the amount of bromelain [30]. Pineapple juice marinade has also improved the tenderness of various parts of spent chicken meat and increased consumer acceptability. The author suggests marinating as a technique to increase the economic value of spent chicken meat [46].

The results obtained in this study showed a reduction in hardness between 6% and 41%. Several authors have observed a decrease in the Warner–Bratzler shear force of beef between 7% and 21% in beef meat with the application of several proteases, including bromelain [18,31]. In another study, the action of bromelain on meat tissue also increased tenderness. The steak meat samples was incubated at room temperature (25  ±  3  °C) for 2 h and treated with purified core bromelain showed a greater reduction (52.1%) in the Warner–Bratzler shear force than samples treated with commercial stem bromelain (26.8%), although both samples showed a reduction in the Warner–Bratzler shear force compared to the control samples [31]. In the present study, the beef meat was marinated with the bromelain present in the pineapple core by-products without extraction or purification. These marinades took place at refrigerated temperatures (5 ± 1 °C) for longer periods of time (0–24 h) and showed a reduction maximum of 41% in Warner–Bratzler shear force compared to the control samples. Although the refrigeration temperature does not approach the optimum temperature of the bromelain activity, the meat’s hardness showed a considerable reduction.

Resistance depends on the quantity of intramuscular connective tissue, intramuscular fat, and sarcomere length. The action of proteolytic enzymes on myofibrillar proteins promotes a decrease in meat firmness due to the breakdown of these proteins into small peptides or proteins with low molecular weight [30,56]. Toughness is instigated by enhanced cross-linking in connective tissue [57]. The integrity of the structure of connective tissues and myofibrils can influence meat softness [12].

Inactivation of bromelain (75 ± 1 °C) may not be complete with medium or rare degrees of cooking; there is the possibility of high residual enzymatic activity after cooking, which can cause over-tenderization [13].

The optimum conditions for bromelain to have maximum activity are temperatures in the range 37–70 °C [17]. Meat quality legislation does not allow storage at temperatures above 7 °C [58]. Although the marinade process was at a temperature below the favorable conditions for the enzyme (4 °C), bromelain showed advantageous results in tenderizing the steaks.

Histology allows the detection of specific tissue components and architectural changes promoted by distinct types of meat processing [59]. Immersion treatments change the structure of steak meat tissue, and the distance between cells increases with decreasing pH. The analysis of histological images showed that the swelling of the meat tissue is due to the increase in the extracellular space or to the swelling of the meat fibers [50]. Another study with bovine muscle notes that the microstructure undergoes profound changes during acidification, but that it regains normal structure when the pH is adjusted to the common pH of raw bovine muscle. This disorganization of muscle proteins and the absorption of water promote a decrease in shear strength and an increase in steak meat tenderization [28]. Steak *Longissimus lumborum* muscle samples immersed in the sonicated papain solution also presented larger intracellular spaces and cell wall disruption, the most evident changes being in the surface layer of the meat [26]. 

The marinating and cooking processes did not improve the yield since the increased water retention (4%) during marinade was lost during cooking (19%), presenting total losses of 15%. The hydration of the meat is due to the infiltration of the marinade in extracellular spaces [60]. The qualitative changes promoted by cooking suggest gradual modifications in the denaturation of collagen fibers, increasing softness, in a study with female carabeef (buffalo) meat. Higher temperature and longer cooking time reduced the shear force value and collagen content, and increased pH, cooking loss, collagen solubility, and tenderness values [61]. Comparable results were found for bovine Semitendinosus muscle increased when roasted at 80–90 °C and soluble collagen amount in bovine meat roasted to 80 °C [62].

## 5. Conclusions

The addition of dehydrated pineapple by-products enriched in bromelain via high hydrostatic pressures to marinades induced positive modifications on meat characteristics. Pineapple by-products were acidic and lower the pH of meat. The lower pH of steak during cooking induced a greater precipitation of proteins with greater loss of yield in cooking. The color of the steaks was more influenced by marinating time than the concentration of bromelain. Long marinades and the addition of bromelain decreased the steaks’ hardness. Skeletal muscle fibers appeared to be more affected by marinating time than by the concentration of bromelain. Severe structural changes occurred due to heat treatment. The intermuscular collagen fibers presented a moderate degree of degradation, regardless of the marinade conditions.

Longer marinades (12–24 h) with a higher concentration of bromelain (10–20 mg tyrosine.100 g^−1^ meat) reduced pH (8%) and hardness (41%) of the steaks, increased marination yield (4%), and turned them lighter in color (L* marinated steak increased 38%) compared to samples without marinade. The pineapple by-products are a source of bromelain and, thus, this work contributes both to the recovery of an agro-industrial waste and to the economic valorization of steak pieces with less commercial value due to their hardness.

## Figures and Tables

**Table 1:** Experimental design with coded and decoded values of independent variables (bromelain concentration and time of contact) for steak samples and dependent variables: pH, marination yield, cooking yield, color, and hardness. (C) means the central point.

Run	Coded Factors		Decoded Factors		pH			Marination Yield (%)	Cooking Yield (%)	Colour									Hardness Steak (N)
	Time	Concentration	Time (h)	Concentration (mg Tyrosine. 100 g^−1^ Meat)	Brine	Marinated Steak	Cooked Steak			Initial			Marinated Steak			Cooked Steak			
										L*	a*	b*	L*	a*	b*	L*	a*	b*	
**8**	0.00000	1.41421	12	20	4.69 ± 0.18	5.05 ± 0.01	5.83 ± 0.03	2.38 ± 0.37	69.31 ± 0.76	38.48 ± 1.12	23.57 ± 1.08	7.23 ± 0.76	51.24 ± 1.99	11.72 ± 1.35	4.27 ± 1.43	56.67 ± 1.48	7.72 ± 0.69	8.75 ± 0.68	32.54 ± 3.91
**9 (C)**	0.00000	0.00000	12	10	4.81 ± 0.04	5.11 ± 0.02	5.84 ± 0.03	2.68 ± 0.29	67.70 ± 0.25	38.20 ± 1.20	24.50 ± 1.28	7.48 ± 1.15	48.89 ± 2.23	12.92 ± 1.35	3.73 ± 0.60	56.80 ± 1.53	7.33 ± 0.47	8.83 ± 0.72	35.50 ± 4.70
**2**	−1.00000	1.00000	3.5	17	4.31 ± 0.03	5.29 ± 0.10	5.65 ± 0.03	1.48 ± 0.08	68.08 ± 0.49	38.27 ± 1.92	25.05 ± 1.58	7.17 ± 1.10	45.08 ± 1.41	15.01 ± 0.76	3.75 ± 0.54	54.51 ± 0.90	7.85 ± 0.29	8.82 ± 0.51	38.36 ± 3.19
**4**	1.00000	1.00000	20.5	17	4.60 ± 0.06	4.99 ± 0.05	5.73 ± 0.01	1.97 ± 0.46	68.23 ± 1.95	38.40 ± 0.82	24.37 ± 1.19	7.34 ± 0.72	49.90 ± 3.13	11.94 ± 1.58	5.93 ± 1.12	58.53 ± 0.78	6.83 ± 0.41	8.77 ± 0.32	30.35 ± 2.50
**10 (C)**	0.00000	0.00000	12	10	4.93 ± 0.01	5.18 ± 0.01	5.84 ± 0.03	3.53 ± 0.87	66.27 ± 0.05	38.91 ± 1.63	24.27 ± 2.18	7.33 ± 1.59	54.11 ± 4.02	11.27 ± 0.78	4.35 ± 1.91	55.90 ± 4.86	8.20 ± 1.84	10.01 ± 0.42	35.47 ± 4.40
**7**	0.00000	−1.41421	12	0	5.61 ± 0.05	5.39 ± 0.07	5.99 ± 0.03	3.58 ± 0.16	69.64 ± 0.84	39.23 ± 1.28	24.88 ± 1.37	7.86 ± 1.22	51.22 ± 2.30	12.72 ± 1.29	4.26 ± 0.40	55.98 ± 1.27	8.44 ± 0.69	10.73 ± 0.44	48.14 ± 4.99
**5**	−1.41421	0.00000	0	10	4.14 ± 0.03	5.34 ± 0.06	5.77 ± 0.03	0.07 ± 0.09	71.18 ± 1.98	37.52 ± 1.08	23.57 ± 0.68	5.87 ± 0.96	39.24 ± 0.51	21.21 ± 1.47	5.75 ± 0.72	50.65 ± 1.68	10.32 ± 1.33	9.57 ± 0.39	41.30 ± 8.58
**6**	1.41421	0.00000	24	10	4.86 ± 0.05	5.07 ± 0.06	5.81 ± 0.05	4.19 ± 0.42	63.13 ± 1.92	38.54 ± 1.66	25.71 ± 2.97	8.26 ± 2.55	52.11 ± 3.80	11.47 ± 0.68	5.18 ± 1.52	59.63 ± 0.93	6.42 ± 0.37	9.18 ± 0.32	36.16 ± 6.81
**11 (C)**	0.00000	0.00000	12	10	4.88 ± 0.01	5.12 ± 0.09	5.80 ± 0.04	2.95 ± 0.63	64.33 ± 0.95	39.30 ± 2.40	25.42 ± 2.59	8.46 ± 1.61	49.70 ± 6.45	13.55 ± 1.72	5.31 ± 1.51	59.45 ± 0.85	6.72 ± 0.26	9.42 ± 0.46	37.14 ± 11.02
**12 (C)**	0.00000	0.00000	12	10	4.89 ± 0.16	5.14 ± 0.02	5.81 ± 0.06	3.54 ± 0.74	66.44 ± 0.46	39.93 ± 0.90	25.25 ± 2.88	8.75 ± 1.63	53.60 ± 0.39	11.05 ± 1.92	4.60 ± 1.10	59.45 ± 1.93	6.54 ± 0.91	8.59 ± 0.65	37.03 ± 8.04
**1**	−1.00000	−1.00000	3.5	3	5.16 ± 0.03	5.34 ± 0.04	5.77 ± 0.08	2.01 ± 0.12	67.21 ± 0.29	39.61 ± 1.66	24.34 ± 1.63	7.22 ± 1.72	46.45 ± 0.51	16.03 ± 0.91	4.34 ± 0.89	55.56 ± 1.00	7.72 ± 0.44	9.80 ± 0.56	43.10 ± 7.60
**3**	1.00000	−1.00000	20.5	3	5.34 ± 0.06	5.21 ± 0.03	5.86 ± 0.01	3.97 ± 0.51	67.47 ± 0.76	38.64 ± 1.17	25.31 ± 1.46	7.73 ± 1.33	50.34 ± 3.80	11.93 ± 1.13	4.74 ± 0.97	58.60 ± 0.73	7.41 ± 0.94	10.25 ± 0.54	38.02 ± 7.83

**Table 2:** Regression coefficients of second-order polynomial equations for each response variable: pH of brine, marinated steak, cooked steak; marination yield (MY); cooking yield (CY); color of marinade and cooked steak; and hardness (H).

Parameter		Equation	R^2^	R^2^ adj	Lack of Fit
**pH**	**Brine**	pH = 5.111* + 0.077 t* − 0.002 t^2^* − 0.115 C* + 0.003 C^2^* + 0.0005 tC	0.97	0.94	Not significant
	**Marinated steak**	pH = 5.476* − 0.016 t* + 0.0004 t^2^ − 0.021 C* + 0.0008 C^2^* − 0.0007 tC	0.95	0.92	Not significant
	**Cooked steak**	pH = 5.820* + 0.017 t − 0.0005 t^2^ − 0.016 C + 0.0004 C^2^ − 0.00006 tC	0.68	0.42	Significant
**Marination yield (MY)**		MY = 0.275 + 0.375 t* − 0.008 t^2^ + 0.059 C − 0.003 C^2^ − 0.006 tC	0.86	0.75	Not significant
**Cooking yield (CY)**		CY = 66.180* − 2.746 t + 0.706 t^2^ + 0.290 C + 2.969 C^2^ − 0.053 tC	0.55	0.18	Not significant
**Color**	**Marinated steak**	Δ*L* = 1.858 + 1.267 t* − 0.036 t^2^* + 0.125 C − 0.005 C^2^ − 0.0007 tC	0.84	0.71	Not significant
		Δ*a* = −2.836 − 1.123 t* + 0.027 t^2^* − 0.185 C + 0.002 C^2^ + 0.011 tC	0.86	0.74	Not significant
		Δ*b* = −0.484 − 0.421 t* + 0.012 t^2^ − 0.094 C + 0.001 C^2^ + 0.009 tC	0.57	0.21	Not significant
	**Cooked steak**	Δ*L* = 12.630* + 0.507 t* − 0.009 t^2^ + 0.246 C − 0.009 C^2^ − 0.0006 tC	0.85	0.73	Not significant
		Δ*a* = −13.691* − 0.330 t + 0.006 t^2^ − 0.232 C + 0.010 C^2^ + 0.004 tC	0.53	0.14	Not significant
		Δ*b* = 4.468* − 0.223 t + 0.007 t^2^ − 0.240 C + 0.009 C^2^ − 0.0007 tC	0.53	0.13	Not significant
**Hardness steak (H)**		H = 48.916* - 0.398 t + 0.009 t^2^ − 1.064 C* + 0.030 C^2^ − 0.012 tC	0.89	0.80	Not significant

*: Parameter significantly affecting the response variable, *p* < 0.05.

**Table 3:** Histological technique images collected through optical microscope with digital camera as a function of marinades and cooking: bromelain concentration (mg tyrosine.100 g^−1^ meat) and time (h).

Sample Processing	Concentration (mg Tyrosine. 100 g^−1^ Meat)	Time (h)	Microphotography	Qualitative Description
Raw	10	12	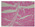	On this microphotograph is visible a moderate degree of disorganization of skeletal muscle fibers and a moderate degree of disorganization of the inter-muscular collagen fibers (H&E. Barr 200 µm).
	17	3.5	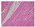	On this microphotograph is visible a slight degree of disorganization of skeletal muscle fibers. (H&E. Barr 200 µm).
		20.5	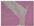	On this microphotography is visible a moderate degree of disorganization of the inter-muscular collagen fibers and muscle fibers are generally preserved (H&E. Barr 200 µm).
	20	12	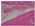	On this microphotography is visible a moderate degree of disorganization of the inter-muscular collagen fibers and muscle fibers are swollen (H&E. Barr 200 µm).
Cooked		12	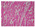	On this microphotograph is visible a severe degree of disorganization of skeletal muscle fibers and a complete disorganization of the inter-muscular collagen fibers (H&E. Barr 200 µm).
